# Impact of surfactant administration through a thin catheter in the delivery room: A quality control chart analysis coupled with a propensity score matched cohort study in preterm infants

**DOI:** 10.1371/journal.pone.0208252

**Published:** 2018-12-12

**Authors:** Pauline Berneau, Trang Nguyen Phuc Thu, Patrick Pladys, Alain Beuchée

**Affiliations:** 1 Department of Pediatrics, University of Rennes 1,Rennes, France; 2 LTSI, University of Rennes 1, Rennes, France; 3 Division of Neonatology and CIC-0203, Department of Pediatrics, CHU Rennes, France; Centre Hospitalier Universitaire Vaudois, FRANCE

## Abstract

**Introduction:**

Most infants born before 30 weeks gestational age (GA) develop respiratory distress syndrome soon after birth. Methods of surfactant administration that avoid ventilation have been recently introduced. The aim of this study was to evaluate the impact of implementing a new procedure of less invasive surfactant administration (LISA) and determine whether it is associated with an improvement in respiratory outcome.

**Methods:**

This single center cohort quality improvement study analyzed preterm infants born before 30 weeks GA between May 2010 and April 2016. Changes in health care practices and respiratory outcomes following the implementation of a LISA, *i*.*e*. the administration of surfactant through a thin catheter, were analyzed using quality control charts. Then, the effect of LISA on respiratory outcome was assessed by propensity score matching and logistic regression weighted by the inverse of the propensity score.

**Results:**

During the study period, 379 infants were included. Of those that were not intubated at ten minutes of life, 129 received surfactant and were ventilated for one hour or more (InVent), 127 received LISA, five received surfactant with tracheal mechanical ventilation for less than one hour (InSurE), and 55 were only treated with nasal continuous positive pressure during the first hour of neonatal care (nCPAP). Quality-chart analysis revealed rapid implementation of the method with a concomitant decrease in required ventilation. LISA was associated with fewer tracheal ventilation days and a lower incidence of supplemental oxygen on day 28. When controlling for the propensity to be exposed or not to LISA, this procedure was not associated with a lower risk of death or bronchopulmonary dysplasia (BPD) at 36 weeks postmenstrual age.

**Conclusion:**

In this study, the successful implementation of the new method was associated with lower rates of mechanical ventilation, but without a significant reduction of grade I/II/III BPD or death.

## Introduction

Advances in neonatal care have resulted in improved survival rates of premature infants. However, there has been limited progress in reducing the rate of bronchopulmonary dysplasia (BPD). The pathogenesis of BPD is multifactorial and includes defects in the synthesis and secretion of surfactant associated with prematurity and ventilator-induced barotrauma and volotrauma [1,2]. Administration of exogenous surfactant has been shown to decrease mortality and the complications of respiratory distress syndrome in premature infants [3].

Recent studies have demonstrated that surfactant can be delivered intratracheally without traditional intubation. These methods include intratracheal surfactant instillation with the help of a thin catheter, aerosolized administration, pharyngeal administration, and laryngeal mask-guided administration. The expected benefits are a reduction in invasiveness and exposure to mechanical ventilation and associated side effects [1,2,4–11].

We introduced surfactant administration through a thin catheter without ventilation (new method) in May 2012 in the delivery room of the neonatal intensive care unit (NICU) at the university teaching hospital in Rennes, France, in accordance with the literature and as part of a quality improvement initiative to reduce the incidence of BPD in preterm infants. The aim of this study was to evaluate whether the introduction of surfactant without ventilation in our regular clinical practice resulted in improved respiratory outcomes. The chosen method for this retrospective analysis was to combine quality control chart analysis with a propensity score matched cohort study to obtain a good view of the variations in clinical practices and outcomes over time.

## Patients and methods

### Setting and subjects

This retrospective, single-center, observational study was conducted between May 1, 2010 and April 31, 2016 in the NICU at the tertiary-care, university teaching hospital of Rennes. This center takes care of premature newborns from 23 weeks GA in a 30-bed ICU. Initial respiratory care took place in the delivery room next to the ICU. All inborn preterm infants with a gestational age (GA) < 30 weeks were included in this study. Infants with congenital malformations and out-born patients, *i*.*e*. those born outside the university teaching hospital of Rennes, at home, or during maternal transport were not included.

The ethics committee of the Rennes University Hospital approved the study and waived the requirement for informed consent. All data were fully anonymized.

### Procedure

Initial resuscitation at birth followed the Neonatal Resuscitation Program guidelines [12]. All preterm infants received continuous positive airway pressure (CPAP) with a face mask and pressure-limited T-piece resuscitator, with the continuous positive pressure generally set at 5 to 6 cm H_2_0 during and following initial resuscitation at birth. Spontaneously breathing preterm infants who had respiratory distress, *i*.*e*. with clinical symptoms of moderate respiratory distress with a fraction of inspired oxygen (FiO_2_) greater than 0.3 for a SpO_2_ between 90 and 95%, received surfactant administration according to the local protocol. Before May 2012, the administration of surfactant was followed by prolonged tracheal positive-pressure ventilation. Early extubation for nasal CPAP in the hour following intubation (InSurE) was rare [4]. The main reasons given were: waiting for reversion of the effects of analgesia-sedation, waiting for stabilization of the respiratory status, and favoring contact of the newborn with its parents [13]. LISA was introduced to the unit on May 21, 2012 and progressively implemented thereafter. This major change in practice was supported by a video demonstration and accompaniment of each practitioner for their first procedures. Within eight months, all practitioners had been trained to perform the procedure and the sedation protocol was consolidated, *i*.*e*. 20 μg/kg atropine associated with 0.5 mg/kg ketamine, repeated once if necessary, preferentially via peripheral venous access [14]. Spontaneously breathing preterm infants on CPAP for whom surfactant therapy was indicated, were intubated via a thin catheter (Vygon^TM^ suction catheter: 1.5 mm in diameter (04Fr) x 265 mm in length) introduced through the nose and guided to the trachea with the help of a Magill forceps under direct laryngoscopy. Immediately after placement of the catheter under direct visualization, the mask on the face was replaced and 6 cm H_2_0 CPAP applied. Surfactant (200 mg/kg) was then slowly delivered through the catheter, over 1 to 3 min, with pauses if cough or desaturation occurred. The catheter was removed immediately after the administration was completed, whereas CPAP was continued with a T-piece and mask and the infant carefully monitored for apneas and SpO_2_ to adapt the respiratory support and O_2_ supply. The target for SpO2 was 92% with alarms set between 88 and 95% all along this study.

### Data recording

Maternal and neonatal data, collected retrospectively from the medical record, included antenatal steroid use, timing of the rupture of the membranes, mode of delivery, birth weight, GA, sex, amount and time of postnatal surfactant administration, and data on adverse events until death or 36 weeks GA.

### Definitions

Four strategies of respiratory care were used in the first hour after birth: (i) intubation with tracheal ventilation for 1 h or more (InVent), (ii) intubation with surfactant administration and tracheal ventilation for less than 1 h (InSurE), (iii) the less invasive surfactant administration (LISA), and (iv) and nasal CPAP with no surfactant administration during the first hour of neonatal care (nCPAP).

Treatment failure was defined as the need for a second dose of surfactant therapy or intubation or re-intubation beyond the first two postnatal hours, regardless of the respiratory care provided at birth.

The primary outcome of the study was survival without moderate to severe bronchopulmonary dysplasia (BPD) at 36 weeks postmenstrual age (PMA) or discharge, defined as grade I to III following the latest revisited BPD definition provided by National Institute of Child Health and Human Development (NICHD) Consensus group [15].

The other respiratory outcomes were the number of days on mechanical ventilation, duration of the first invasive ventilation period, need of mechanical ventilation on day 3, post-natal age at O_2_ withdrawal, and need of supplementary oxygen at day 28.

The secondary outcomes were the incidence of pneumothorax, grade 3 or 4 intraventricular hemorrhage, cystic periventricular leukomalacia, surgical treatment of patent ductus arteriosus (PDA), surgery for necrotizing enterocolitis (NEC) and late onset sepsis. NEC was classified according to Bell’s modified classification [16]. We have constructed a composite outcome “death or major morbidities” that includes grade 3 or 4 intraventricular hemorrhage, cystic periventricular leukomalacia, retinopathy of prematurity in indication of laser therapy and grade I/II/III BPD. Since the method for screening ROP has changed, we added a second composite outcome not including severe ROP.

The side effects commonly associated with the technique, such as bradycardia, apnea, or desaturation, were not systematically collected. However, tolerance to the procedure was assessed on 46 patients during its implementation and appeared to be safe (5% apnea-bradycardia, 6% unbalanced administration of surfactant, and frequent surfactant reflux).

### Statistical analysis

All data were completely anonymous. Categorical data are presented as n (%). Continuous data were tested for normality (Q-Q plots and Shapiro tests) and are presented as medians [25p, 75p]. Proportion per units of the new procedure, the duration of first tracheal mechanical ventilation associated with surfactant therapy, total duration of tracheal mechanical ventilation, and death or BPD at 36 weeks PMA were sequentially analyzed over time using *xbar-*charts and *u*-charts as appropriate [17]. The evaluation period was divided into successive 4-month periods, from May 2010 (period 1) through April 2016 (period 24) for analysis of the control charts. Upper (UCL) and lower (LCL) control limits were set at ±3σ. Changes in the processes were determined according to Shewhart rules [18]. The mean gestational age, z-Score and duration of first tracheal ventilation associated with surfactant therapy were analyzed over time using Exponential Weighted Moving Average (EWMA) charts. Similarly, the proportion per units of the LISA procedure and intubation before min 10 were sequentially analyzed over time using *u*-charts for all the population. The proportion per units of death and grade I/II/III BPD were then sequentially analyzed over time using *u*-charts for the infants not intubated at min 10.

Subsequently, we used a propensity score approach [19] to control for observed confounding factors that could influence group assignment, *i*.*e*. new or InVent procedure for surfactant administration in spontaneously breathing infants after 10 minutes of life (min 10). The propensity score was estimated using a logistic regression model with LISA as the dependent variable in relation to the following baseline characteristics: GA, gender, birth weight z-score, multiple birth, antenatal corticosteroids, hypertension during pregnancy, prolonged rupture of membranes, maternal infection/chorioamnionitis, mode of delivery, Apgar score, post-natal age at intubation, and early neonatal infections. The propensity score was then used as a distance measure to perform 1:1 matching with replacement. The population size was set at 500 for optimization by the evolutionary algorithm [19]. Imbalance after matching was checked. Differences between the two groups, exposed, or not, to LISA were examined with χ2 tests or Fisher's exact tests for categorical data and independent t-tests or Mann-Withney U-tests, as appropriate, for continuous data. Then, odds ratios (ORs) were calculated to quantify the association between the initial strategy and main outcome using logistic regression fit by generalized estimating equations, weighted by the inverse of the propensity score. The best model was selected according to its parsimony and performance by stepwise selection with the AIC, BIC, and LASSO penalty methods. A two-tailed p value < 0.05 was considered statistically significant for all analyses. Data were analyzed with R software (R Core Team (2014). R: A language and environment for statistical computing. R Foundation for Statistical, Computing, Vienna, Austria. URL http://www.R-project.org/).

## Results

### General characteristics of the population

**Fig 1:**
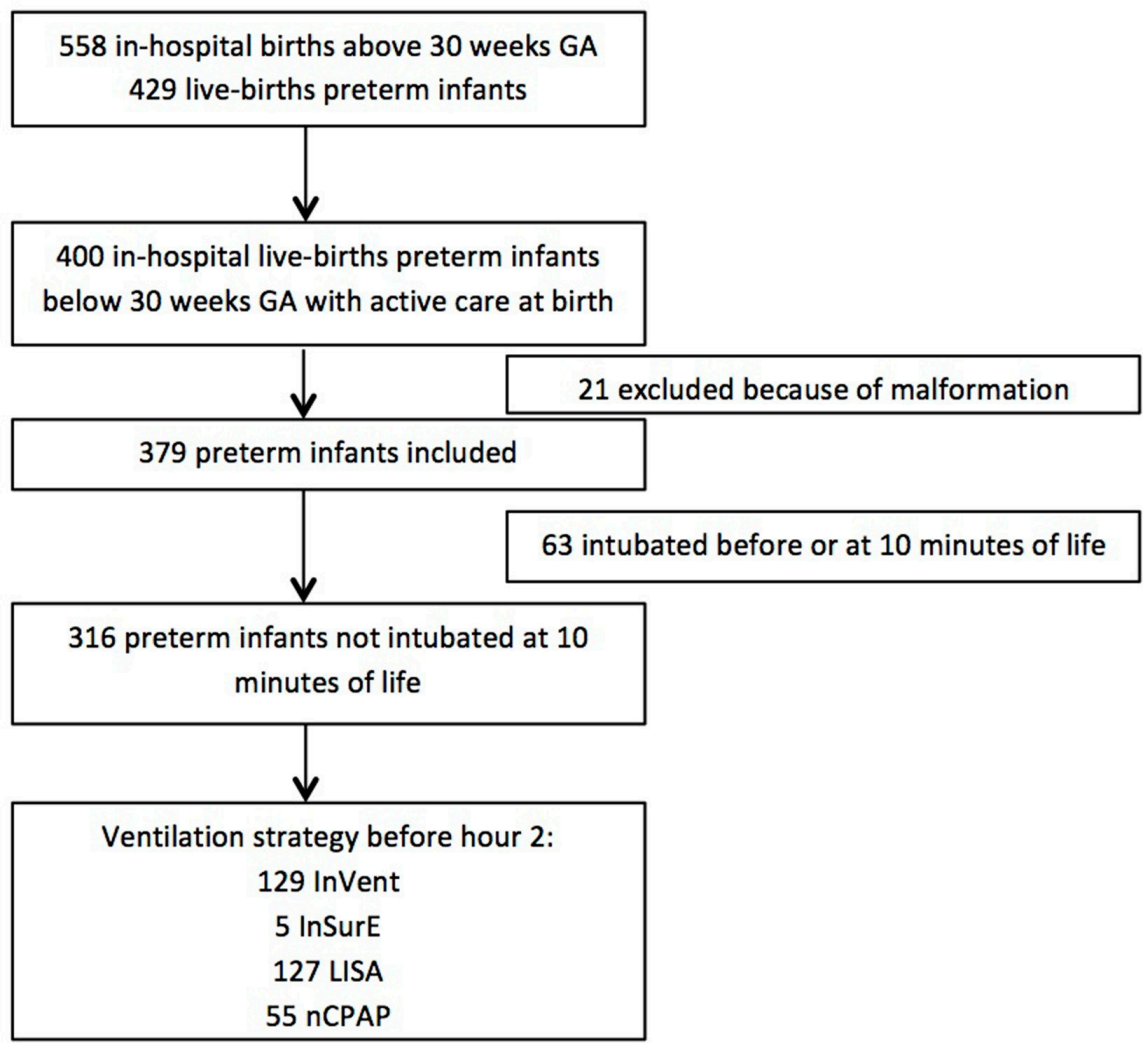
Flow chart. InVent: intubation with tracheal ventilation for 1 h or more. InSurE: intubation with surfactant administration and tracheal ventilation for less than 1 h. LISA: less invasive surfactant administration of surfactant. nCPAP: no surfactant administration during the first 2 hours of neonatal care.

From May 2010 to April 2016, 400 patients were born alive below 30 weeks GA and had active care at birth in the NICU. Of the eligible infants, 21 were excluded because of congenital malformations. The initial respiratory support is described in the flow chart (Fig 1). One patient in the InVent group, one with the new procedure, and three in the CPAP group were transferred to another medical center before 36 weeks PMA or discharged with missing data in their follow-up.

The demographic and clinical characteristics of the population are shown in Table 1. The infants intubated before or at min 10 received less prenatal corticosteroids and were more frequently exposed to chorioamnionitis (p < 0.05), had lower birthweight and GA (p < 0.01), and had lower 1- and 5-min Apgar scores (p < 0.001) than spontaneously breathing infants at min 10. Similarly, they had higher rates of death or major morbidities (Table 1). The incidence of grade I/II/III BPD at 36 weeks PMA in the surviving infants was not significantly different between infants intubated before or at min 10 (63) and spontaneously breathing infants at min 10 (316), despite a significant difference in the initial respiratory support (62/63 *vs* 129/316 InVent, p < 0.001) and subsequent respiratory outcomes (32/56 *vs* 84/315 for tracheal ventilation on day 3, p < 0.001 and five days [1; 22] vs one day [0; 8] of mechanical ventilation, p < 0.001).

**Table 1:** Demographic and clinical data of the overall population.

	Not intubated at min 10 N = 316	Intubated at min 10 N = 63	p	N
Gestational age (weeks)	28.3 [27.0;29.1]	27.0 [25.7;28.1]	<0.001	379
Birthweight (g)	1060 [849;1226]	910 [788;1090]	0.007	379
Birthweight (z Score)	-0.02 [-0.52;0.56]	0.09 [-0.42;0.61]	0.592	379
Boys	174 (55.1%)	35 (55.6%)	1	379
Multiple births	104 (32.9%)	26 (41.3%)	0.258	379
Pre-eclampsia	85 (26.9%)	11 (17.5%)	0.157	379
Preterm Labor	195 (61.7%)	46 (73.0%)	0.119	379
Premature rupture of membranes	76 (24.1%)	16 (25.4%)	0.947	379
Chorio-amnionitis	62 (19.6%)	21 (33.9%)	0.021	378
Prenatal steroids	290 (91.8%)	49 (77.8%)	0.002	379
Caesarean section	208 (65.8%)	39 (61.9%)	0.652	379
1-min Apgar Score	7.00 [5.00;8.25]	2.00 [1.00;3.50]	<0.001	379
Respiratory support at birth (before hour 2):			<0.001	379
InSurE	5 (1.58%)	1 (1.59%)		
InVent	129 (40.8%)	62 (98.4%)		
LISA	128 (40.5%)	0 (0.00%)		
nCPAP	54 (17.1%)	0 (0.00%)		
Surfactant therapy	276 (87.3%)	62 (98.4%)	0.018	379
Duration of first ventilation associated with surfactant administration	0.25 [0.00;24.0]	47.9 [17.9;192]	<0.001	325
Mechanical ventilation	208 (65.8%)	57 (90.5%)	<0.001	379
Mechanical ventilation on day 3	84 (26.7%)	32 (57.1%)	<0.001	371
Duration of mechanical ventilation (days)	1.00 [0.00;8.00]	5.00 [1.00;22.0]	<0.001	377
Any respiratory support (mechanical ventilation or CPAP) (days)	43.0 [25.0;65.0]	42.0 [16.2;79.5]	0.769	374
Air leak	8 (2.54%)	9 (15.5%)	<0.001	373
Supplemental O2 at 28 days postnatal age	227 (77.2%)	39 (83.0%)	0.486	341
Grade I/II/III BPD	113 (44.1%)	21 (51.2%)	0.209	327
Early Onset Sepsis	2 (0.64%)	2 (3.45%)	0.116	372
Late Onset Sepsis	68 (21.8%)	11 (20.4%)	0.956	366
Grade 3 or 4 intraventricular haemorrhage	30 (9.68%)	16 (29.1%)	<0.001	365
White matter damage	34 (10.8%)	14 (22.2%)	0.022	379
Cystic periventricular leukomalacia	10 (3.16%)	0 (0.00%)	0.380	379
Surgical treatment of necrotising enterocolitis or focal intestinal perforation	7 (2.24%)	3 (5.56%)	0.170	367
Laser of retinopathy of prematurity	11 (3.89%)	4(9.52%)	0.114	325
Surgical treatment of patent ductus arteriosus	32 (10.3%)	9 (16.7%)	0.252	366
Death before discharge	24 (7.67%)	22 (34.9%)	<0.001	376
Death or grade I/II/III BPD	137 (43.9%)	43 (68.3%)	0.001	375
Death or major morbidities	146 (46.9%)	45 (71.4%)	0.001	374
Death or major morbidities including ROP	152 (49.4%)	45(71.4%)	0.002	371

Data are presented as n (%) or the median [1^st^ quartile; 3^rd^ quartile]. InVent: intubation with tracheal ventilation for 1 h or more; InSurE: intubation with surfactant administration and tracheal ventilation for less than 1 h; LISA: less invasive surfactant administration; nCPAP: no surfactant administration during the first 2 h of neonatal care.

The composite outcome “death or grade I/II/III BPD” was less frequent in spontaneously breathing infants than in those who were intubated before or at M10 (p = 0.002). The technique failed within the first 72 h for 35 infants (27.6%) of the LISA group, 20 (36.4%) of the nCPAP group, and one (20%) of the InSurE group, and the infants required intubation or reintubation.

### Changes in clinical practice in the delivery room

**Fig 2:**
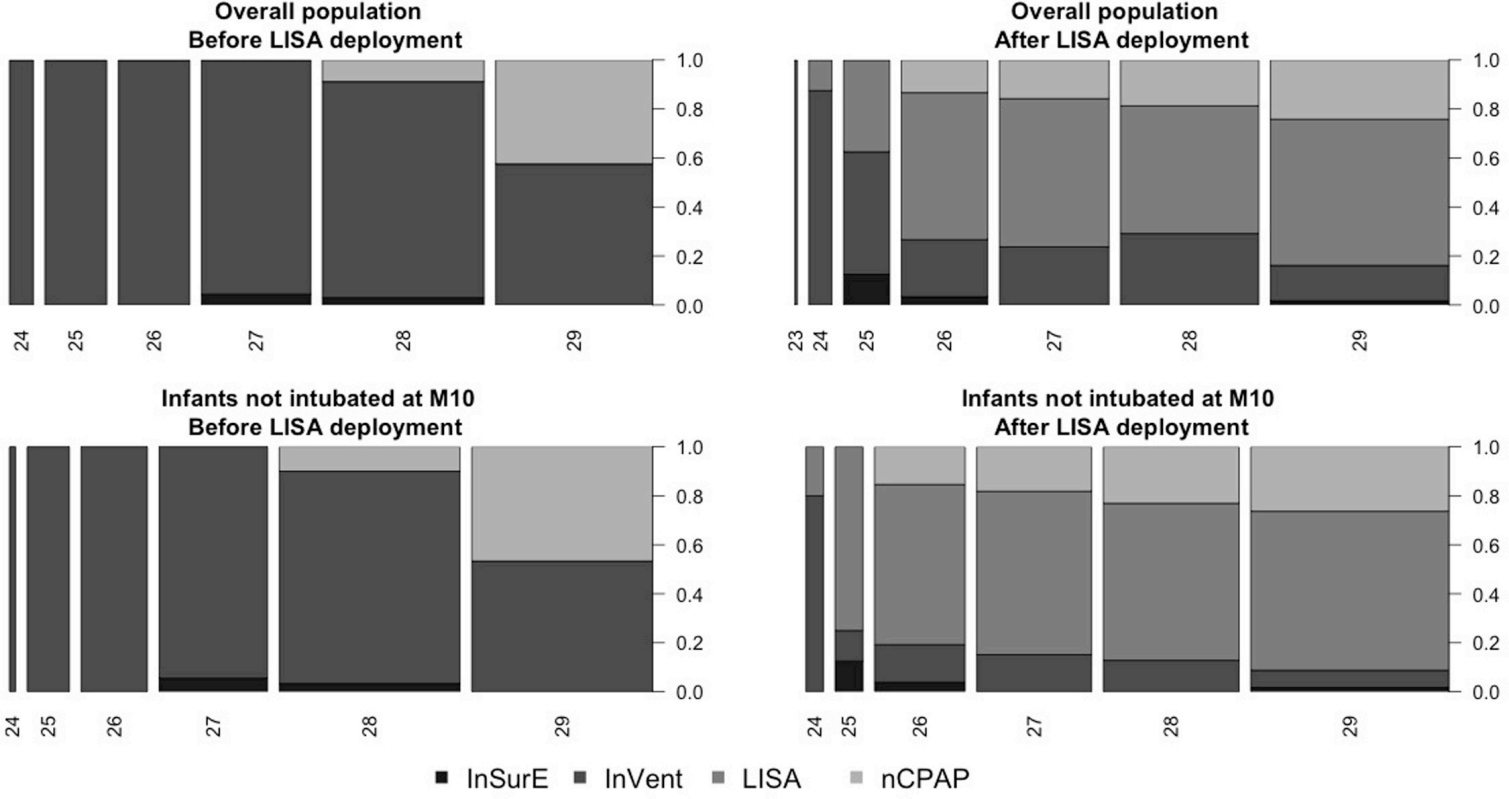
Evolution of ventilation strategies after LISA deployment. Ventilation strategies used in the delivery room according to GA at birth for the overall population before and after LISA deployment. Ventilation strategies used in the delivery room according to GA at birth for the group of preterm infants who were not intubated at 10 min postnatal age before and after LISA deployment.

The ventilation strategy used in our delivery room changed after LISA deployment in the unit essentially for the infants not intubated at min 10 (Fig 2).

**Fig 3:**
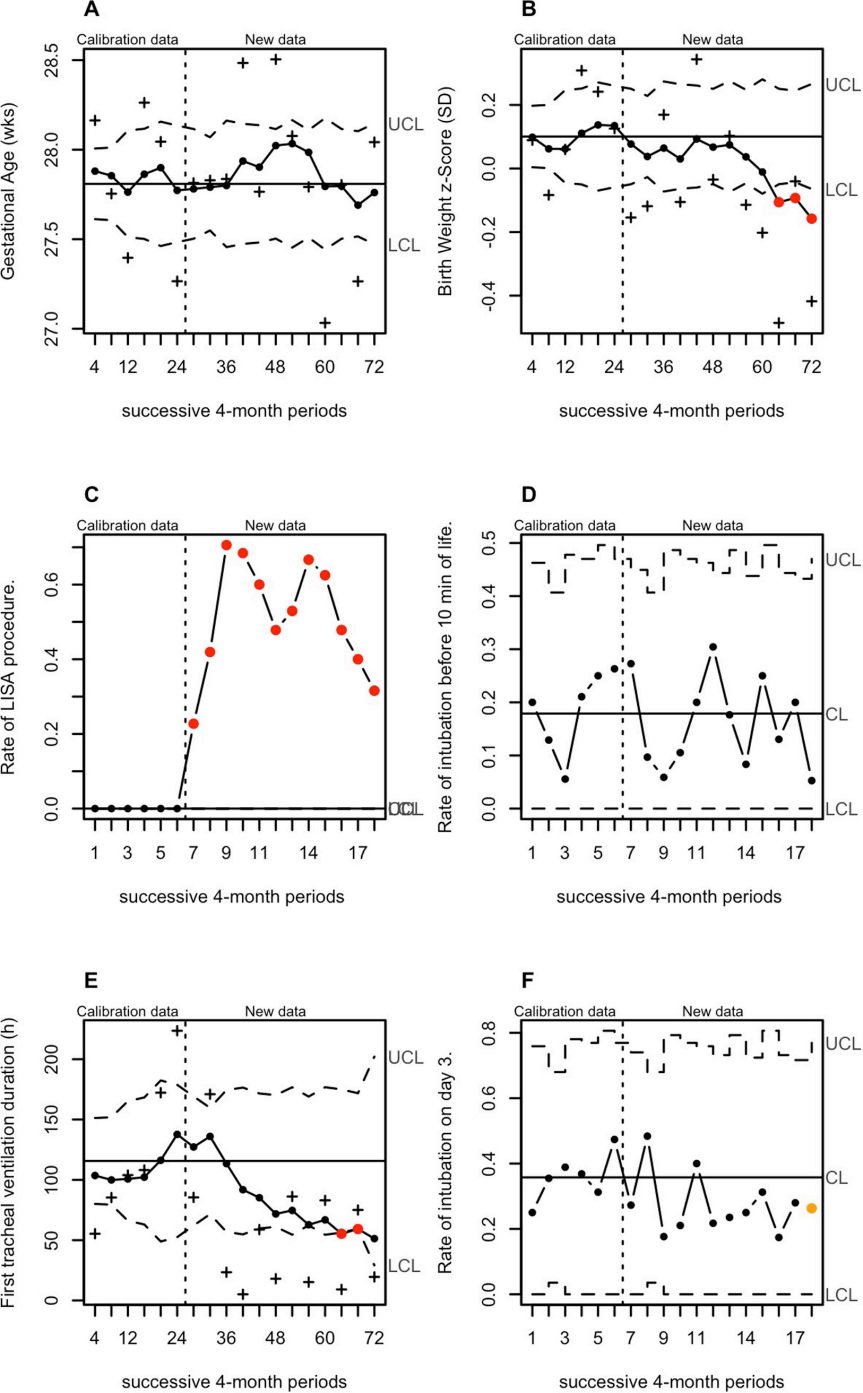
Analysis of successive 4-month periods through quality-control charts in the overall population. Analysis of the trend for GA at birth (A), z-Score of the birth weight (B), the proportion of LISA (C), the rate of intubation at 10 minutes of life (D), the duration of first tracheal ventilation (E) and the proportion of tracheal ventilation on day 3 (F). Violated runs according to Shewhart rules are indicated by the red (large shift above 3 SD) and orange (small sustained shift) dots, indicating a significant change in the trend of the process. UCL and LCL designate the upper and lower control limits. Graphics A, B and E were EWMA-charts and graphics C, D and F were u-charts. The (+) in Fig 3A, 3B and 3E- refer respectively to the gestational age, birth weight z-Score and duration of first tracheal ventilation averages for each successive 4-month periods. The (●) in Fig 3A, 3B and 3E- refer to the moving average of series of data with weights, which decay exponentially.

Analysis of the 18 consecutive four-month periods through quality-control charts highlights the changes in our practices in the delivery room and the respiratory outcome during the implementation of LISA (Fig 3). Approximately 50% of patients received surfactant without ventilation in the delivery room in the eight months following introduction of the technique in the unit. One year later, approximately 70% of all preterm infants received surfactant without ventilation in the delivery room. This change in our practices was associated with a sustained shift in short-term respiratory outcome (duration of the first mechanical ventilation associated with surfactant administration and the proportion of mechanical ventilation on day 3) but no significant change in the rate of death or grade I/II/III BPD at 36 weeks PMA. At the same time, the profile of the overall population noticeably changed, with a shift in the trend of the birth-weight z-Score towards lower values 72 months after introduction of the procedure.

**Fig 4:**
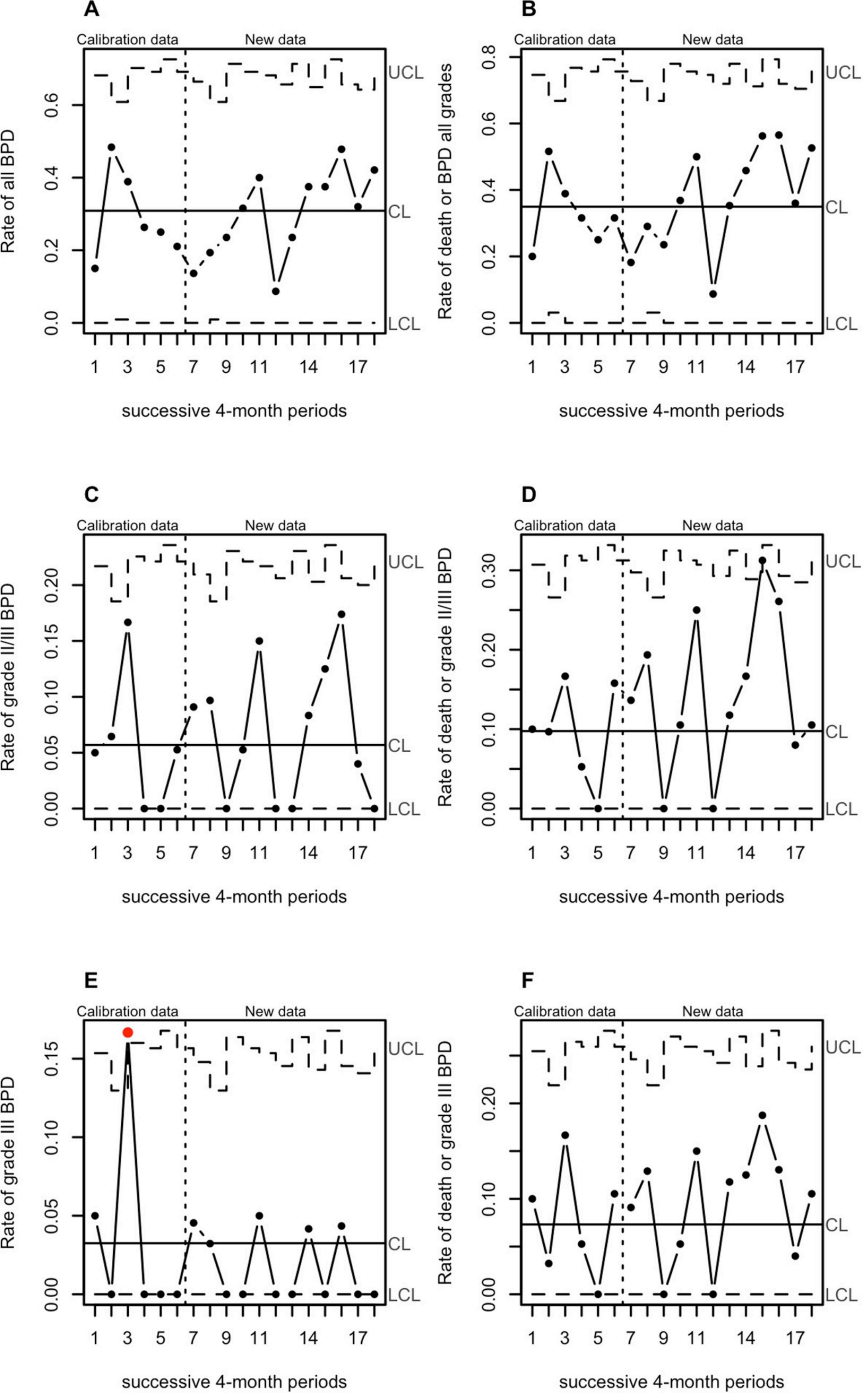
Changes in rate of death and BPD in spontaneously breathing infants at min 10 through quality-control charts. Rate of all BPD (A), death or BPD all grades (B), grade II/III BPD (C), death or grade II/III BPD (D), grade III BPD (E), and death or grade III BPD (F), for each of successive 4-month periods.

The rate of the primary outcome “death or grade I/II/III BPD” varied in the range of a stable process during the study period for the infants in spontaneously breathing infants at 10 min of life (Fig 4).

### Effect of LISA on survival without BPD

Among the 261 spontaneously breathing infants at min10 with an indication for surfactant, 127 treated with the new procedure could be matched with 127 infants treated with InVent. The five infants exposed to InSurE were not included in the matching procedure. The matched groups were found to be well-balanced for prenatal and early neonatal characteristics at birth (Table 2).

**Table 2:** Data from the antenatal period after matching with propensity score.

	InVent N = 127	LISA N = 127	p
Gestational age (weeks)	28.1 [27.3;29.1]	28.1 [27.0;29.2]	0.925
Birthweight (g)	1040 [880;1150]	1045 [832;1238]	0.979
Birthweight (z-Score)	0.05 [-0.47;0.44]	-0.08 [-0.56;0.53]	0.982
Boys	68 (53.5%)	68 (53.5%)	1.000
Multiple births	36 (28.3%)	36 (28.3%)	1.000
Pre-eclampsia	44 (34.6%)	44 (34.6%)	1.000
PROM	30 (23.6%)	30 (23.6%)	1.000
Chorio-amnionitis	17 (13.4%)	17 (13.4%)	1.000
Use of antenatal steroids	119 (93.7%)	119 (93.7%)	1.000
Caesarean section	85 (66.9%)	86 (67.7%)	1.000
APGAR	7.00 [5.00;8.00]	7.00 [5.00;8.00]	0.876

Data are presented as n (%) or the median [1^st^ quartile; 3^rd^ quartile]. InVent: intubation with tracheal ventilation for 1 h or more; LISA: less invasive surfactant administration; PROM: premature rupture of membranes

After matching with propensity score, the univariate comparison both group of the incidence of death or grade I/II/III BPD did not show a significant difference (p = 0.6) (Table 3). The incidence of the grade III BPD was significantly less in the LISA group.

**Table 3:** Primary outcome after matching with propensity score.

	InVent N = 127	LISA N = 127	p.
Death:			
Death before day 7	4 (3.15%)	1 (0.79%)	0.370
Death before discharge	7 (5.74%)	7 (5.56%)	1.000
BPD discrete:			0.069
No BPD	67 (5.8%)	75 (63.0%)	
Grade I BPD	35 (30.2%)	34 (28.6%)	
Grade II BPD	4 (3.45%)	8 (6.72%)	
Grade III BPD	10 (8.62%)	2 (1.69%)	
Grade I/II/III BPD	49 (42.6%)	44 (37.3%)	0.487
Grade II/III BPD	14 (12.2%)	10 (8.47%)	0.476
Grade III BPD	10 (8.70%)	2 (1.69%)	0.034
Death or BPD:			
Death or grade I/II/III BPD	56 (45.9%)	51 (40.5%)	0.463
Death or grade II/III BPD	21 (17.2%)	17 (13.5%)	0.524
Death or grade III BPD	17 (13.9%)	9 (7.14%)	0.124

Data are presented as n (%). InVent: intubation with tracheal ventilation for 1 h or more; PROM: premature rupture of membranes; LISA: less invasive surfactant administration; GA: gestational age; BPD: bronchopulmonary dysplasia; PMA: postmenstrual age.

### Effect of LISA on other respiratory outcome

Infants who received the LISA procedure had a lower duration of mechanical ventilation and a lower incidence of supplemental oxygen required on day 28 (Table 4).

**Table 4:** Other respiratory outcomes after matching with propensity score.

	InVent N = 127	LISA N = 127	p.
Post-natal age at intubation	30.0 [20.0;35.0]	30.0 [25.0;39.0]	0.205
Surfactant therapy (no. of doses):			0.783
1	120 (94.5%)	117 (93.6%)	
2	6 (4.72%)	7 (5.60%)	
3	0 (0.00%)	1 (0.80%)	
6	1 (0.79%)	0 (0.00%)	
Duration of first ventilation associated with surfactant administration	23.6 [13.4;47.5]	0.00 [0.00;0.00]	<0.001
Secondary intubation	29 (22.8%)	56 (44.4%)	<0.001
Duration of mechanical ventilation (days)	2.00 [1.00;9.00]	0.00 [0.00;5.00]	<0.001
Tracheal mechanical ventilation on day 3	30 (23.6%)	23 (18.1%)	0.354
Supplemental O_2_ at age 28 days	103 (85.8%)	82 (68.3%)	0.002
Any respiratory support (mechanical ventilation or CPAP) (days)	43.0 [32.0;64.5]	42.0 [18.5;63.5]	0.086
Post natal age at O_2_ withdrawal (week’s GA)	35 [33.0;37.1]	34.3 [31.6;36.7]	0.110

Data are presented as n (%) or the median [1^st^ quartile; 3^rd^ quartile]. InVent: intubation with tracheal ventilation for 1 h or more; PROM: premature rupture of membranes; LISA: less invasive surfactant administration; GA: gestational age; BPD: bronchopulmonary dysplasia; PMA: postmenstrual age

### Effect of LISA on non-respiratory outcome

The incidence of late onset sepsis, pneumothorax, necrotizing enterocolitis, PDA, severe intraventricular hemorrhage, and periventricular leukomalacia and survival without major complications did not significantly differ between groups (Table 5).

**Table 5:** Secondary outcomes after matching with the propensity score.

	InVent N = 127	LISA N = 127	P
Early Onset Sepsis	0 (0%)	1 (0.79%)	1.000
Late Onset Sepsis	28 (22.0%)	33 (26.6%)	0.486
Pulmonary Late Onset Sepsis	17 (13.4%)	21 (16.7%)	0.579
Catheter-days	13.0 [9.5;20.5]	12.0 [9.00;22.0]	0.242
PDA	45 (35.4%)	34 (27.0%)	0.189
Surgery for PDA	12 (9.60%)	11 (8.73%)	0.984
Surgery for NEC or focal intestinal perforation	1 (0.79%)	4 (3.20%)	0.211
Air leak	1 (0.79%)	4 (3.17%)	0.213
White matter damage Cystic periventricular leukomalacia	11 (8.66%) 2 (1.57%)	11 (8.66%) 5 (3.94%)	1.000 0.447
Death before day 7	4 (3.15%)	1 (0.79%)	0.370
Death before discharge	7 (5.74%)	7 (5.56%)	1.000
Death or major morbidities	59 (48.4%)	56 (44.8%)	0.665

Data are presented as n (%) or the median [1^st^ quartile; 3^rd^ quartile]. LISA: less invasive surfactant administration; InVent: intubation with prolonged tracheal ventilation; PDA: patent ductus arteriosus; NEC: necrotizing enterocolitis; HIV: intraventricular hemorrhage.

Multivariate analysis with sensitivity analysis of the overall population was consistent with the primary analysis based on propensity matching. After adjustment for GA, birth weight z-score, and late onset sepsis, LISA was significantly associated with a reduction in the risk of death or grade I/II/III BPD at 36 weeks PMA (OR(95% CI) = 0.55 (0.31–0.97); p < 0.05). This association was no longer significant after weighting by the inverse of the propensity score (OR (95% CI) = 0.66 (0.3–1.04); p = 0.07). GA and birth weight z-score were the only variables significantly associated with more survival without grade I/II/III BDP.

## Discussion

This cohort quality improvement study demonstrates the feasibility and clinical benefit of implementing a new procedure in a single level 3 neonatal unit, with training and accompaniment. The administration of surfactant through a thin catheter, without mechanical ventilation, could be successfully applied during the first hour after birth for infants younger than 30 weeks GA. This procedure was associated with a lower initial and overall duration of tracheal mechanical ventilation, lower duration of all respiratory support, and lower incidence of all respiratory support on day 28. When controlling for the propensity to be exposed or not to LISA, this procedure was not associated with a lower risk of death or grade I/II/III BDP at 36 weeks PMA or discharge in our population, whereas some recent meta-analyses of LISA techniques did report such an effect [6,20,21].

BPD is still the most frequent adverse outcome for infants born less than 30 weeks GA, despite the introduction of antenatal steroids, postnatal surfactant, modern respiratory care, and improved nutrition [22,23]. The prevalence of BPD has increased, along with the increase in survival of infants born before 28 weeks GA [24], with rates that remain high, approximately 40%, over the last few years [25]. Diverse approaches have been adopted to protect against lung injury and the development of BPD and thus, significant efforts have been made to avoid the use of invasive ventilation. The recent ILCOR 2015 and European Consensus Guidelines [26] recommend giving priority to a gentle respiratory approach, thus avoiding unnecessary mechanical ventilation during neonatal resuscitation.

A recent meta-analysis of 895 infants showed that the use of LISA reduced the composite outcome of death or BPD at 36 weeks, the need of mechanical ventilation within 72 h of birth, and the need of mechanical ventilation anytime during the ICU stay [20]. Another meta-analysis compared seven ventilation strategies for preterm infants younger than 33 weeks GA [6]. LISA was associated with a lower likelihood of the composite outcome of death or BPD at 36 weeks PMA than mechanical ventilation and nasal CPAP alone. Similar results were reported in a third meta-analysis published in 2016, in which LISA resulted in a decreased risk of BPD, death, or BPD and early CPAP failure [21]. These meta-analyses support previous results of randomized controlled trials and large multi-centric cohort studies using various methods of LISA with different catheters, exposition, and sedation [10,27]. The potential common benefits of these techniques are the maintenance of spontaneous breathing and laryngeal function of infants while receiving nasal CPAP during the procedure, the complete avoidance of intermittent positive pressure ventilation via an endotracheal tube, and reduced traumatic and inflammatory airway injuries [3,26–28]. These new methods and procedures have led to the reassessment of practices and new issues. Control charts have been developed as an industrial quality control technique and the use of such monitoring in healthcare settings was advocated in the late 1980s. This statistical procedure has already been used in the monitoring and improvement of diverse areas of hospital performance [29]. Our results are consistent with those of a recent study with the same objective of quality improvement [30]. In this study, a management protocol, including delayed umbilical cord clamping in combination with optimized nCPAP and less invasive surfactant administration, was associated with improved respiratory outcomes. In our study, the main change observed in our clinical practice in the delivery room over the considered period was the implementation of surfactant administration without mechanical ventilation. We did not demonstrate an increased rate of survival without BPD, but observed a significant improvement in all short-term respiratory parameters studied. Here, we examined differences in neonatal characteristics, including infant GA, birth weight, and other confounders, to assess changes over time that might have influenced outcomes. We observed that the incidence of intrauterine growth retardation increased over time. This had to be considered since fetal growth restriction has been independently associated with an increased risk of chronic lung disease and death [31,32].

The introduction of this technique in our unit was a success. Another minimally invasive technique, known since the early 90s, the InSurE procedure, failed to be efficiently implemented in our unit. Before introduction of the new procedure, the duration of first ventilation was high in our unit, with an average of 60 h. It was sometimes difficult to extubate infants during the first hour and numerous factors may had explained the delay in extubation after surfactant administration with the tube that permitted ventilation: waiting for reversion of the effects of analgesia-sedation, waiting for stabilization of the respiratory status, and favoring contact of the newborn with its parents.

The incidence of secondary complications was similar in the LISA and InVent groups. Several studies have reported a reduction in major brain injuries with the LISA technique [11,33,34]. The very low incidence of severe intraventricular hemorrhage in our study could explain the absence of differences between the groups.

Retinopathy of prematurity (ROP) could not be analyzed since the method for the screening has changed during the study period. Before August 2012, the diagnosis of ROP was only performed using binocular indirect ophthalmoscopy and the implementation of wide-field digital retinal imaging (WFDRI) occurred at the same time as the new procedure. This method is known to provide higher specificity and sensitivity [35]. The change with ROP diagnostic methods could have contaminated the data.

The study has other limitations. We cannot exclude that the absence of a significant difference on the primary outcome is not due to a sufficient sample, since we did no sample size calculations for our study. The objective of the study was to observe the changes in our practices and their consequences. Thus, the indication and condition for surfactant administration were not controlled and the matching method only partially controlled for confounding factors, despite rigorous adjustment for them. Indeed, the method only accounts for observed covariates and any hidden bias due to latent variables may remain after matching. In this matching procedure, controls were used as matches for more than one treated individual, *i*.*e*. with replacement, since it can often decrease bias, especially when there are few control individuals relative to the number of treated individuals. However, inference is more complex, since the matched controls are no longer independent [36]. The complementary weighted multivariate analyses performed on all the patients avoids this limitation. The respective indications of the different methods of respiratory support, the optimal surfactant doses and sedation/analgesia associated with the procedure, optimal GA, and risk factors for failure are yet to be determined. In our unit, we used a sedation protocol with ketamine, but some authors did not use sedation [37]. Sedation used for the InSurE, InVent, and LISA procedures might have had a role in the subsequent immediate failure or success and longer respiratory outcome. This cannot be explored in this retrospective study because the absence of sedation was extremely rare, usually because of the absence of venous access and the presence of rapidly progressing respiratory distress, and sedation was insufficiently documented if no adverse event occurred. Additionally, some variables that might influence the association between LISA and respiratory outcome, such as the timing of caffeine administration or cumulative dose, were not collected [38].

## Conclusion

Advances in neonatal care have resulted in increased rates of extremely premature birth, leading to an emerging population of long-term survivors with BPD. This quality improvement study, using control charts together with propensity scores, permitted the evaluation of a new procedure. This new method of surfactant delivery resulted in less need for mechanical ventilation in spontaneously breathing preterm infants with RDS stabilized with nCPAP, without a significant effect on the composite outcome of death or BPD at 36 weeks. Further studies are needed to optimize the technique and its indications and harmonize premedication protocols.
